# Editorial: Fitting Psychometric Models: Issues and New Developments

**DOI:** 10.3389/fpsyg.2017.00856

**Published:** 2017-05-29

**Authors:** Yanyan Sheng

**Affiliations:** Counseling, Quantitative Methods, and Special Education, Southern Illinois UniversityCarbondale, IL, United States

**Keywords:** test theory, psychometric modeling, structural equation modeling, item response theory, model-data fit, parameter estimation

Test theory provides a framework to evaluate the psychometric properties of an instrument, such as item analysis, test development, test-score equating, and differential function analysis. The theory relies on formulating a statistical model to specify the relationship among a set of test concepts while making certain assumptions about these concepts and their relationships. Hence, it is essential to understand the conditions and assumptions that are necessary for an accurate estimation of the model and hence an adequate fit to the data before the application of a test theory and related models.

The year of 1904 where Thorndike published the first book on test theory, *An Introduction to the Theory and Social Measurement*, marks the beginning of the development of classical test theory, which has provided the theoretical foundation for educational and psychological measurement in the twentieth century (Thorndike, 1940). In the past five decades, classical test theory has been rapidly expanded in various directions (Crocker and Algina, 1986). Specifically, as the focus in data analysis is moving from univariate to multivariate procedures, the statistical modeling of test data is becoming more complex involving structural equation modeling (SEM), or modeling with modern test theories such as item response theory (IRT) and generalizability theory. Fitting a complex psychometric model relies on the ability to accurately estimate the model parameters, which can be realized with the availability of enhanced computational technology and the emergence of advanced statistical estimation methods, such as the weighted least squares (WLS), the expectation-maximization algorithm, and the Markov chain Monte Carlo (MCMC) simulation techniques.

This research topic brings together a selection of insightful papers that focus on fitting psychometric models for polytomous or dichotomous responses that often appear in aptitude, achievement, personality, and interest measures used in education and psychology. Specifically, the seven articles published in this research topic demonstrate the current development in classical and modern test theories where more complicated modeling of test data is realized via the use of SEM or IRT, with the focuses being (1) issues and practices associated with fitting or estimation of an existing psychometric model, (2) applications of the test theory and models to real data problems, and (3) proposals of new test models and/or methods that offer advantages not realized with existing ones.

With respect to individual papers, Ropovik and Barendse et al. focused on model-data fit in the context of SEM, with the former calling attention to the χ^2^ model test that has been usually disregarded in applications of latent variable modeling, whereas the latter proposing indices for assessing model-data fit when the analysis is based on pairwise maximum likelihood (PML), which was believed to perform better than WLS for factor analyzing discrete ordinal response data. The pairwise likelihood method belongs to the broad class of pseudo-likelihoods (Besag, 1975; Cox and Reid, 2004), which replaces the likelihood by a function that is easier to evaluate and consequently easier to maximize.

De Bondt and Van Petegem, Guo et al., on the other hand, demonstrated two case studies of applications of SEM to real data problems where the structural validity of questionnaires that measure personality and sleep quality, respectively, was investigated. It is worth noting that instead of using the frequentiest approach, De Bondt and Van Petegem adopted a Bayesian SEM (Lee, 2007; Kaplan and Depaoli, 2012; Muthén and Asparouhov, 2012) method to their problem and found that with informative priors for cross loadings and residual variances the model-data fit was adequate, which was not achieved using the maximum likelihood SEM. Bayesian SEM is an innovative and flexible approach to latent variable modeling, and this paper demonstrates its applications and further the advantages of Bayesian over frequentist in fitting latent variable models via the use of SEM.

Finally, the three articles by Jiang et al., Kuo and Sheng, and Park et al. all involved evaluating parameter estimation in IRT, with the first two focusing on multidimensional graded response models (GRM; Samejima, 1969)—applicable for Likert items with ordered categories, and the latter focusing on an important assumption for conventional dichotomous IRT models. All three articles evaluated performances of the respective model(s) using Monte Carlo simulations and consequently provided a set of guidelines under various test situations that were manipulated by the studies. Specifically, Jiang et al. assessed the performance of the marginal maximum likelihood method (MML) paired with the EM algorithm in estimating the multidimensional GRM [a straightforward extension of the multidimensional IRT (Reckase, 2009) model] under different sample size, test length and intertrait correlation conditions. Kuo and Sheng focused on a special case of the multidimensional GRM, namely, the multi-unidimensional GRM, and compared a number of different MML and fully Bayesian (via the use of MCMC techniques) methods in estimating the model under different test conditions. Park et al., on the other hand, illustrated the effect of item parameter drift on estimating unidimentional IRT models (via the use of mixture models), calling for the importance of checking the invariance assumption before fitting the model.

With current enhanced computational technology and advanced statistical estimation methods, complex modeling of test data can be realized via the use of e.g., PML, WLS, or MCMC under the framework of SEM or IRT. However, each method or modeling has its own set of limitations. Consequently, it is essential that one understands the conditions under which the model fits adequately to ensure that little error and bias are involved in estimating model parameters. When applying psychometric models to real data problems, one needs to keep in mind that all (simple or complex) models are approximations to reality. It is hence important that we evaluate various aspects of model-data fit in order to decide on the best available model, which may not necessarily be the most complicated one. The seven articles in this research topic exemplify evaluating the fit of complex psychometric models empirically or theoretically, which will serve as a good reference for future research on developments and applications of psychometric models. It is hoped that more studies are conducted in this area to further advance test theory.

## Author contributions

The author confirms being the sole contributor of this work and approved it for publication.

### Conflict of interest statement

The author declares that the research was conducted in the absence of any commercial or financial relationships that could be construed as a potential conflict of interest.
